# Isolation and Characterization of Effective Bacteria That Reduce Ammonia Emission from Livestock Manure

**DOI:** 10.3390/microorganisms10010077

**Published:** 2021-12-30

**Authors:** Sun-Il Kim, Wan Heo, So-Jung Lee, Young-Jun Kim

**Affiliations:** 1 Department of Food and Biotechnology, Korea University, Sejong 30019, Korea; sunil_2003@naver.com (S.-I.K.); sjl4859@gmail.com (S.-J.L.); 2 Mediogen, Co., Ltd., Bio Valley 1-ro, Jecheon-si 27159, Korea; 3 Department of Food Science and Engineering, Seowon University, Cheongju 28674, Korea; 01062033526@seowon.ac.kr

**Keywords:** ammonia, emission, fine dust, livestock manure, nitrogen, *Pediococcus acidilactici*

## Abstract

Ammonia from livestock manure reacts with chemical components discharged from various emission sources to produce airborne particulate matter. This study aimed to investigate a novel effective microbial agent to suppress ammonia gas emitted from manure. Both isolated L12I and 12III strains, identified as *Pediococcus acidilactici* (PA), were selected for their superior activity in assays performed with the evaluation criteria such as acid production, ammonia decomposition, and urease inhibition, which are key factors influencing ammonia excretion. The survivability of PA strains was confirmed by an increase in DNA abundance in the manure. PA strains lowered the pH of manure and suppressed the growth of hyper-ammonia-producing bacteria (HAB) possessing urease activity. The L12I and 12III treatment groups showed 23.58% and 38.00% emission reductions, respectively. Especially, the 12III strain was proven to be the more effective strain for reducing ammonia gas emission, with the best ability to reduce pH and inhibit HAB. The strains could have an additive effect in improving the manure quality as a nitrogen fertilizer by preserving the total nitrogen and urea content. These results suggest that PA strains can be used as unprecedented microbial agents to improve manure-derived environmental pollution and improve fertilizer quality.

## 1. Introduction

Recently, it has been reported that the fine dust level in Korea corresponds to a high concentration risk level among countries that have signed the Convention on the Organization for Economic Co-operation and Development (OECD) [1]. Fine dust is a carcinogenic substance that causes various diseases due to oxidative penetration into human organs, such as parts of the respiratory system and skin [2]. Gaseous chemicals emitted from various sources, such as livestock, factories, and automobiles, react with each other and produce fine dust in the atmosphere, and ammonia serves as the main precursor of fine dust. Ammonia reacts with other volatile components such as oxides of nitrogen and sulfur and volatile organic compounds in the atmosphere to produce particulate fine dust, such as ammonium sulfate and ammonium nitrate [3]. In addition, ammonia itself directly irritates human eyes, the respiratory tract, and skin, and prolonged exposure can permanently damage human health [4].

According to recent reports by the European Environment Agency (EEA, EU) and the National Institute for Environmental Research (NIER, Incheon, Korea), the livestock sector accounts for a large proportion of ammonia emissions worldwide, and the field of livestock manure administration is reported to be responsible for about 90% of ammonia emission in the agriculture sector [5,6]. Livestock manure contains various nitrogen compounds including urea, and ammonia accumulates in the manure because of the enzymatic activity (urease and deaminase) of the microflora present in the manure [7,8,9,10]. The accumulated ammonia dissolves in water and increases the pH of the manure owing to the separation of hydroxide ions. The pH elevation correlates to an exponential increase in conversion of ammonia to gaseous form, thereby increasing ammonia emission [11,12].

Several methods that reduce the concentration and atmospheric emission of ammonia have been investigated [13]. Among these methods, microbial application has been shown to result in fewer secondary environmental pollution problems compared to physicochemical methods such as gas barriers, acidifiers, and absorbents [14,15]. However, many studies on biological inhibitors focus on reducing harmful gases such as ammonia through nitrogen metabolism, and studies on the cause of ammonia emission from manure and the interaction between microflora by inhibitors are insufficient [15].

Effective microorganism (EM) products are generally utilized to reduce the odor and promote fermentation in agricultural fields and comprise actinomycetes, *Bacillus subtilis*, lactic acid bacteria (LAB), yeasts, etc. Additionally, extensive research shows that they are known to be effective in reducing various harmful gases [16,17]. *Bacillus subtilis* and actinomycetes present in EM products are known to be effective in reducing the concentration of nitrogen present as ammonia [18,19,20,21]. Moreover, LAB can facilitate acid production and exert antimicrobial activity against pathogens via bacteriocin production [22,23]. Therefore, this study was conducted with the aim of selecting functional microorganisms that reduce pH, decompose ammonia, and inhibit urea hydrolysis, all of which can contribute to reducing ammonia emission. Furthermore, novel applications of microbial agents can be attempted to prevent the spread of contamination derived from agricultural by-products by verifying their activity in the manure.

## 2. Materials and Methods

### 2.1. Isolation, Maintenance, and Identification of Microbial Cultures

EM products were obtained from the Agricultural Technology Center (Sejong, Korea) and Onsiya (Seoul, Korea), and several bacteria were isolated from them. The EM products used in this study are commercially available to reduce harmful agricultural gas emissions and improve livestock diseases and are fermented broths in which LAB, *Bacillus subtilis* and yeasts are co-cultured. The products were serially diluted and spread on plate count agar (PCA, Difco^TM^, Detroit, MI, USA), and the plates were incubated at 35 °C for 24 h to isolate the bacteria. They were isolated on the basis of morphological distinction of colonies and were spread on PCA containing bromocresol purple (BCP-PCA, Eiken Chemical Co., Ltd., Tokyo, Japan). The plates were then incubated at 35 °C for 24 h to isolate LAB that produce acids. Until the bacterial selection process, all isolated bacteria were sub-cultured more than twice in plate count broth (PCB, Difco^TM^) at 35 °C in a microbial incubator (JSR incubator JSRI-250T, Gongju, Korea) prior to each experiment. All media were sterilized at 121 °C for 15 min. The screened strains were identified using the API 50 CHL test (BioMérieux, Lyon, France), which is a biochemical identification kit [24,25,26]. The selected bacteria were cultured using the API 50 CHL kit (BioMérieux) at 30 °C for 48 h. After incubation, the APIWEB software (BioMérieux) was used to analyze the results of fermentation of 49 carbohydrates by the strains.

### 2.2. Changes in the pH during Bacterial Culturing

A pH meter (Orion model A211, Thermo Scientific, Waltham, MA, USA) was used to measure the acid-producing capacity of the isolated strains. The strains were inoculated at 2% (*v*/*v*) in PCB (Difco^TM^) and incubated at 35 °C. The pH of the cultivated medium was measured at 0, 6, 12, and 24 h.

### 2.3. Ammonia Removal from the Liquid Medium

The ammonia degradation by the isolated strains was analyzed by quantifying the ammonia concentration in the enriched culture medium. Ammonium chloride (Fisher Scientific International, Inc., Pittsburgh, PA, USA) was added at a concentration of 1% (*w*/*v*) to the PCB (Difco^TM^), and 2% concentration (*v*/*v*) of the bacterial subculture was inoculated into the medium. Bacterial strains were incubated at 35 °C and shaken at 130 rpm. After 24 h, the culture was centrifuged at 4 °C and 10,000× *g* to obtain the supernatant, and the ammonia concentration in the supernatant was quantified using the indophenol method [27].

### 2.4. Urease Inhibition Activity of the Isolated Strains

The urease inhibition activity of the isolated strains was evaluated using the phenol red method to confirm the change in ammonia production due to urease hydrolysis [28,29,30]. The substrate solution was prepared by dissolving 2% urea (TCI, Tokyo, Japan) and 0.2% phenol red (TCI, Tokyo, Japan) in 0.1 M Tris-HCl buffer (pH 6.8; LPS solution Co., Daejeon, Korea). After adding 20 µL urease from *Canavalia ensiformis* (U1500; Sigma-Aldrich, St. Louis, MO, USA) and 100 µL single culture of isolated strains to 500 µL substrate solution, 380 µL distilled water was added to the reaction mixture, and after incubating the mixture for 30 min at 37 °C, the absorbance was measured at 560 nm.

### 2.5. Manure Samples and the Growth Conditions of Microbes

Livestock manure samples obtained from livestock farms (Anseong, Korea) were used to verify the effectiveness of the selected strains. The samples consisted of 50%, 10%, 10%, and 30% of swine manure, cattle manure, poultry manure, and sawdust, respectively. The L12I and 12III strains were selected and identified as *Pediococcus acidilactici* (PA), were cultured in de Man, Rogosa, and Sharp (MRS, Difco^TM^) broth at 35 °C for 24 h in a JSRI-250T incubator (JSR incubator), and this procedure was utilized for all experiments. *Clostridium aminophilum* (KCTC 5424) and *Proteus mirabilis* (KCTC 2510), which are known as hyper-ammonia-producing bacteria (HAB), were purchased from the Korean Collection for Type Cultures (KCTC; Daejeon, Korea) and used to quantify the bacterial DNA present in the manure. *C. aminophilum* was cultured in reinforced clostridial medium (RCM, DifcoTM) and was incubated in an AnaeroPack purchased from Mitsubishi Gas Chemical Co. (MGC; Tokyo, Japan) at 37 °C. *P. mirabilis* was incubated in nutrient broth (NB, DifcoTM) at 37 °C in a JSRI-250T incubator (JSR incubator).

### 2.6. The Rate of Ammonia Removal in Minimal Salt Medium

To determine whether the selected bacterial strains utilize ammonia as an inorganic nitrogen source, an analysis of the concentration of the ammonia–nitrogen in the M9 minimal medium (MB cell, Seoul, Korea) supplemented with carbon and nitrogen was performed. Glucose (2%, *w*/*v*; Oriental Chemical Industries Co., Ltd., Seoul, Korea) was added as a carbon source, and 1% (*w*/*v*) ammonium chloride (Fisher Scientific International, Inc.) was added as a nitrogen source. Bacterial culture (2%, *v*/*v*) was inoculated into the medium and incubated at 35 °C for 24 h. The microbial cultures were centrifuged at 4 °C and 10,000× *g*, and the supernatant was used for the determination of the concentration of ammonia–nitrogen using the indophenol method [27].

### 2.7. DNA Extraction and Real-Time Quantitative Polymerase Chain Reaction (qPCR) Analysis

**Figure 1:**
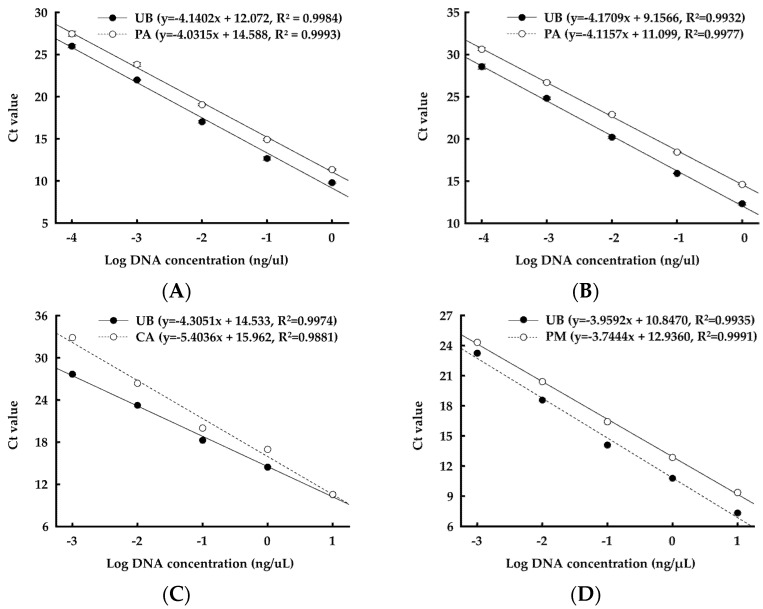
Linear correlation between the log of DNA concentration of microbial single cultures and Ct values from qPCR analysis. Each reaction was performed in triplicate. UB, universal bacteria; PA, *Pediococcus acidilactici*; CA, *Clostridium aminophilum*; PM, *Proteus mirabilis*. (**A**) L12I, (**B**) 12III, (**C**) *Clostridium aminophilum* KCTC 5424, (**D**) *Proteus mirabilis* KCTC 2510.

A Fast-DNA spin kit for soil (MP Biomedicals, Santa Ana, CA, USA) was used to extract DNA. The concentration of extracted DNA was quantified using a NanoDrop ND-1000 (Thermo Scientific). Primers required for qPCR analysis are summarized in Table 1 [31,32,33,34]. The method described by Bokulich et al. [35] was modified for qPCR amplification in this study. Amplification was carried out in a total volume of 20 μL of the mixture, consisting of 10 μL of 2 × GoTaq^®^ qPCR master mix (Promega, Wisconsin, WI, USA), 1 μL of each forward and reverse primer (10 pmol), and 8 μL of DNA template (2.5 ng/μL) using a 7500 real-time PCR system (Applied Biosystems, Waltham, MA, USA). The reaction was run for 45 cycles: pre-denaturation at 95 °C for 10 min, denaturation at 95 °C for 15 s, and annealing and extension at 60 °C for 1 min. The melting curve analysis was conducted at 95 °C for 15 s, 60 °C for 1 min, and 95 °C for 30 s. The DNA extracted from the single microbial cultures was serially diluted, and standard curves for quantification were generated by plotting the cycle threshold (Ct) values for diluted DNA concentrations obtained from the qPCR analysis (Figure 1).

### 2.8. Abundance of the Selected Strains in Manure under Different Culture Conditions

Genomic analysis of the selected bacterial strains in manure was performed to confirm the abundance of the selected strains in manure. To homogenize the sample, 1 g of the manure sample was mixed with 5 mL of distilled water. A total of 160 μL of single culture of the selected strain was inoculated at a low-level concentration of 7.98 log colony forming units (CFU) and a high-level concentration of 8.58 log CFU. The changes in the DNA proportion were analyzed after 24 h of incubation. Aerobic culture conditions were provided by incubating the culture at 35 °C in a shaking incubator at 130 rpm, and anaerobic conditions were provided by using the MGC AnaeroPack and incubating the culture at 35 °C.

### 2.9. Determination of pH Change and Growth of HAB in Manure

For the analysis of pH change and HAB growth due to microbial treatment, 100 μL of single culture was inoculated into a solution containing 50 mL distilled water and 10 g livestock manure. After incubating the culture at 35 °C for 24 h under aerobic and anaerobic conditions, the pH of the manure was measured, and the DNA concentrations of *P. mirabilis* and *C. aminophilum* were measured by qPCR analysis.

### 2.10. Monitoring Ammonia Gas Emission and the Chemical Properties of Manure

Ammonia emissions from manure were analyzed using an air trapping system modified from the method described by Park et al. [36]. For the analysis of ammonia emission from manure after microbial treatment, 1 mL of single microbial culture was inoculated with 100 g of manure placed in the chamber of the device. Ammonia emitted in the chamber was directed into a gas-bubble-collecting flask containing 50 mL 0.05 N H_2_SO_4_ (95%; Dae Jung Chemicals, Gyeonggi, Korea) via air inflow using an air pump and a gas flow meter, and the ammonia was trapped at the same time. Air inflow and outflow rates were maintained at 1 L/min to analyze daily ammonia emission. The collected ammonia was quantified using the indophenol method [27].

The changes in environmental parameters of the samples on days 0 and 35 were analyzed by chemical characterization: pH, organic matter (OM), total nitrogen (T-N), and urea concentration. The pH, OM, and T-N were analyzed by referring to the method described in detail by the Rural Development Administration (RDA, Jeonju, Korea) [37,38], and in detail, OM was analyzed by the direct ash method and T-N by the Kjeldahl method. Urea concentration was quantified using the diacetyl-monoxime method described by Rahmatullah et al. [39].

### 2.11. Statistical Analysis

Each experimental result was expressed as the mean ± standard error of the mean of triplicate experiments, and statistical analysis was performed using the SAS v.9.4 program (SAS Institute Inc., Cary, NC, USA). Differences between the groups were confirmed by Student’s *t*-test and one-way ANOVA, followed by Duncan’s post hoc test with a significance level of *p* < 0.05.

## 3. Results and Discussion

### 3.1. Screening and Identification of Potential Effective Bacterial Strains for Reducing Ammonia Emission

**Figure 2:**
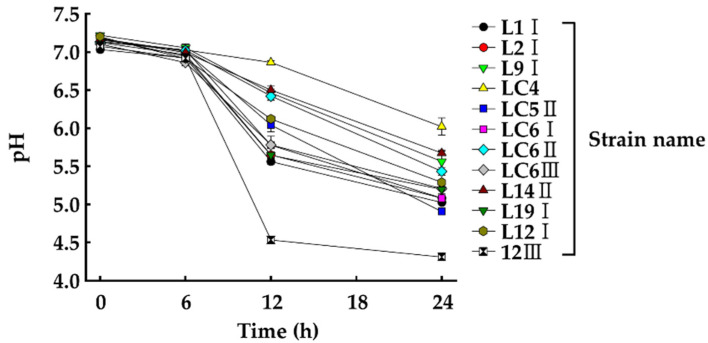
Changes of pH in culture medium of candidate bacterial strains. Values represent the mean ± S.E. (*n* = 3).

Sixty-seven bacterial strains were isolated from commercial EM, among which 12 acid-producing bacteria were selected based on colony color change to yellow in BCP-PCA. Further, the pH changes during incubation of these strain cultures for 24 h were monitored (Figure 2). As a result, eight strains were confirmed to produce acid by decreasing the pH level by 2 or more. Among the strains tested, 12III showed the best acid-producing ability.

**Figure 3:**
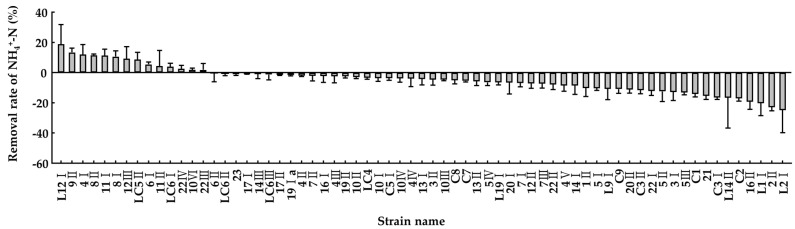
Ammonia removal rate of candidate bacterial strains in liquid medium with NH_4_Cl by using indophenol method. Values represent the mean ± S.E. (*n* = 3).

The potential strains, capable of removing ammonia–nitrogen, were screened based on analysis of the ammonia removal rate during incubation. Ammonia removal ability was tested using 67 isolated bacteria (Figure 3).

**Figure 4:**
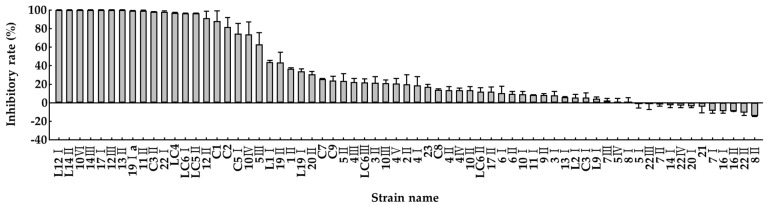
Inhibitory effects of liquid culture of candidate bacterial strains against the urease activity by using phenol red method at 560 nm. Values represent the mean ± S.E. (*n* = 3).

Consequently, all eight selected strains could remove more than 10% of the total ammonia, and L12I removed approximately 20% of the total ammonia. Moreover, a urease activity inhibition assay was conducted to select effective bacterial strains that inhibit urease activity, a major factor of ammonia release (Figure 4). Approximately 20 bacterial strains inhibited urease activity by more than 50%, among which 14 strains showed 100% inhibition.

### 3.2. Identification of Selected Strains by Using API 50 CHL Test

L12I and 12III bacterial strains that exhibited all the required properties such as pH reduction, ammonia degradation, and urease inhibition were finally selected as potentially effective microbes that could reduce ammonia emissions from manure. The selected L12I and 12III strains were both identified as *Pediococcus acidilactici* (PA) with a similarity of 99.9% (Table 2). PA is a probiotic microorganism that is resistant to a wide range of temperatures and pH [40], and it is known to have excellent antibacterial effects due to the production of bacteriocin [40]. The selected strains are expected to be effective in inhibiting ammonia emission from manure on the basis of the screening processes and previously reported activities.

### 3.3. Ammonia Removal Capacity of the Isolated Bacteria in the Minimal Salt Medium

**Figure 5:**
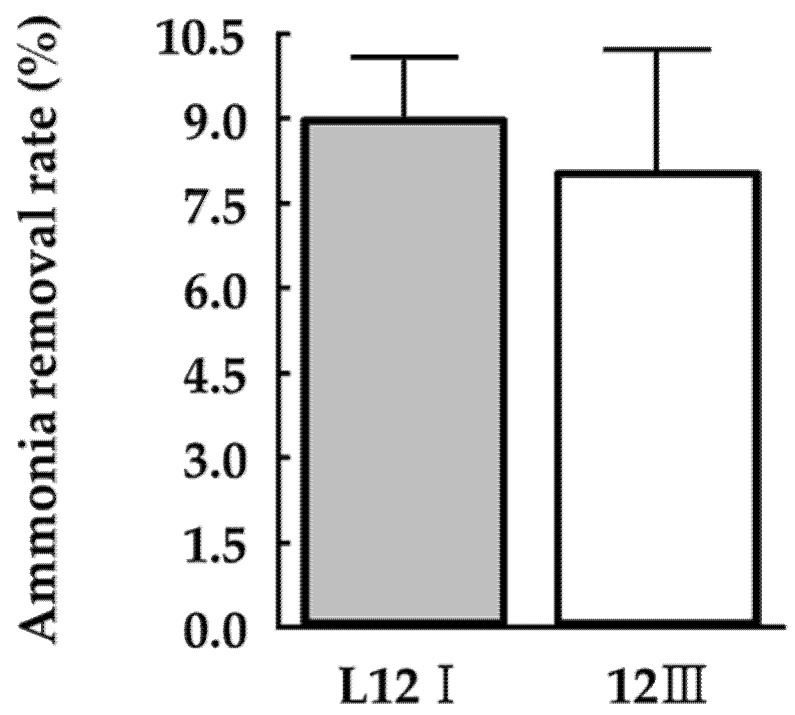
Ammonia removal rate of selected bacterial strains in the minimal medium by using indophenol method. Values represent the mean ± S.E. (*n* = 3).

Bacterial nitrogen metabolism has been modulated to reduce the emission of ammonia by nitrification, denitrification, or nitrogen fixation [18,21,41]. The ability of selected bacterial strains to utilize the nitrogen stored as ammonia was tested in the M9 minimal medium to limit possible metabolic disturbances due to medium components (Figure 5). As a result, PA strains showed the capacity to remove approximately 10% of the nitrogen stored as ammonia; however, the difference in ammonia removal rate between the strains was not significant (*p* > 0.05). Additionally, the ammonia emitted from the medium was not detected. PA can also produce amino acids from inorganic nitrogen sources [42,43]. In this respect, this result suggests that reduced amount of ammonia–nitrogen was not volatilized but instead utilized by PA strains.

### 3.4. DNA Abundance of Selected Strains in Manure

**Figure 6:**
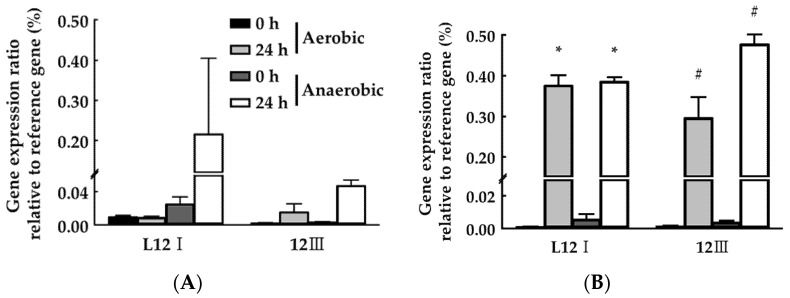
Quantitative real-time PCR analysis to determinate DNA proportion of selected bacterial strains in manure. (**A**) Low-level inoculation (7.98 log CFU), (**B**) high-level inoculation (8.58 log CFU). Values represent the mean ± S.E. (*n* = 3). * *p* < 0.05 (0 vs. 24 h of L12I under the same culture conditions), and # *p* < 0.05 (0 vs. 24 h of 12III under the same culture conditions) indicates statistical significance.

The changes in the PA strains’ DNA abundance in manure under different culture conditions was determined to confirm that the selected bacteria are the predominant type among all the bacteria in the manure (Figure 6). Since the livestock manure composting operations are performed by agitation or sedimentation [44], the survivability of the selected strains depended on the presence of oxygen. The survivability of the PA strains in manure was confirmed. Furthermore, the DNA of PA was not detected in the manure sample not treated with PA strains. The DNA abundance ratio after incubation compared to the initial of PA strains increased depending on the inoculation concentration and showed a significant increase under all culture conditions (Figure 6B). PA strains are thought to be active under both aerobic and anaerobic conditions [45,46], and have the potential to predominate among the microflora in the manure.

### 3.5. Changes in pH and Growth Inhibition of HAB in Manure by the Selected Strains

Ammonia accumulation in the manure dissociates hydroxide ions, resulting in an increase in the pH of the manure. The pH level is closely related to the conversion of ammonia to the gaseous phase, and an increase in pH promotes the release of gaseous ammonia [11,47]. As such, changes in pH and ammonia concentration are essential factors affecting ammonia gas emission. According to recent research reports, iron chloride, sulfuric acid, and other acidic chemicals have been applied to reduce ammonia emissions by modulating the ammonia concentration and gaseous phase conversion [8,48,49]. In this study, it was confirmed that the selected strains (L12I, 12III) were effective in reducing the pH and inhibiting urease activity through a screening process [40,50].

A liquid manure medium was used for a more accurate observation of the pH level changes, and the selected bacterial strains were treated under aerobic and anaerobic conditions (Table 3).

As a result, the pH level of the untreated control was significantly elevated when observed at 0 h compared to the sample under aerobic conditions. In contrast, the pH level of the groups treated with selected strains showed a tendency to decrease. In the case of anaerobic culture conditions, in contrast to aerobic conditions, the pH of all groups tended to reduce after 24 h of incubation. Among them, the groups treated with PA strains showed a significant decrease in pH compared to the control (*p* < 0.05). The reversal pattern between the aerobic and anaerobic control groups could be a phenomenon due to the metabolism of aerobic ammonia producing bacteria in the manure.

**Table 3:** Change of pH in manure by using selected bacterial strains in aerobic and anaerobic conditions. (L) Low-level inoculation (7.98 log CFU), (H) high-level inoculation (8.58 log CFU).

Time (h)			0		24	
Conditions	Group		Average	SE	Average	SE
Aerobic	Control		7.22	0.03	7.34 *	0.04
	L12I	(L)	7.28	0.02	7.25	0.02
		(H)	7.26	0.06	7.31	0.01
	12III	(L)	7.25	0.03	7.27	0.03
		(H)	7.23	0.04	7.21	0.05
Anaerobic	Control		7.24	0.09	6.94 ^a,^*	0.03
	L12I	(L)	7.28	0.05	6.56 ^b,^*	0.01
		(H)	7.22	0.03	6.53 ^b,^*	0.02
	12III	(L)	7.20	0.01	6.55 ^b,^*	0.01
		(H)	7.22	0.03	6.52 ^b,^*	0.01

Values represent the mean ± S.E. (*n* = 3). * *p* < 0.05 vs. 0 h indicates statistical significance. The different letters (a, b) indicate statistically significant difference between different groups at the same time and same conditions (significance level at *p* < 0.05).

**Figure 7:**
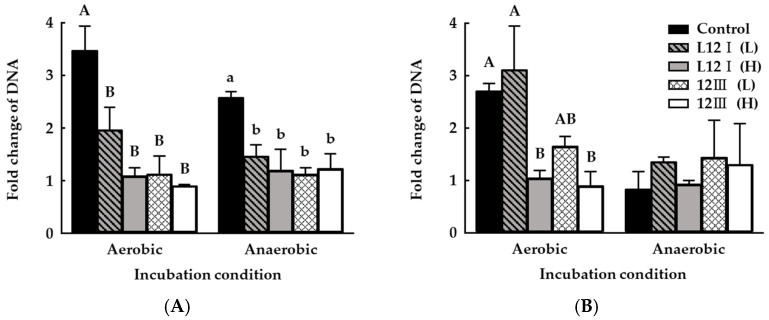
Inhibitory effects of selected bacterial strains against *Clostridium aminophilum* (**A**) and *Proteus mirabilis* (**B**). (L) Low-level inoculation (7.98 log CFU), (H) high-level inoculation (8.58 log CFU). Values represent the mean ± S.E. (*n* = 3). The different capital letters (A, B) indicate statistically significant difference between different groups under the aerobic conditions (significance level at *p* < 0.05). The different minuscule letters (a, b) indicate statistically significant difference between different groups under the anaerobic conditions (significance level at *p* < 0.05).

Ammonia producing bacteria via urea hydrolysis are present in livestock manure [9,10,51]. Furthermore, the DNA of *C. aminophilum* and *P. mirabilis*, representative HAB strains with urease activity [9,10], was present in the manure used in this study. The DNA content of *C. aminophilum*, an obligate anaerobe, increased by more than twofold under all culture conditions (Figure 7A), and the DNA of *P. mirabilis*, an obligate aerobe, increased only under aerobic conditions by approximately threefold (Figure 7B). Overall, all PA strains significantly decreased the change in DNA fold change of *C. aminophilum* compared to the controls under all culture conditions (*p* < 0.05). Likewise, PA strains tended to inhibit the DNA increase of *P. mirabilis* compared to the untreated control under aerobic conditions, and especially, the 12III strain showed a significant inhibition against growth of *P. mirabilis* even in the low inoculation concentration group (*p* < 0.05 vs. control under the aerobic conditions in Figure 7B by using *t*-test).

In our study, it was suggested that the inhibitory effects against HAB affected pH (Table 3), which was reduced by PA treatment and was similar to the inhibition against HAB (Figure 7). PA is well known for its antimicrobial-peptide-producing capacity [22,40] and has been verified to have positive effects on acid production and urease inhibition (Figure 2 and Figure 4). Therefore, it was expected that PA strains could have a positive effect on the inhibition of ammonia emission due to their inhibitory effect on HAB proliferation through pH control and urease inhibition. Furthermore, the results suggested that decrease in ammonia emission is not only a result of the chemical reaction that occurs but is also due to the major influence of various metabolisms of the microorganisms on ammonia emission.

### 3.6. Ammonia Emission from Manure and Changes in the Chemical Properties of the Manure

**Figure 8:**
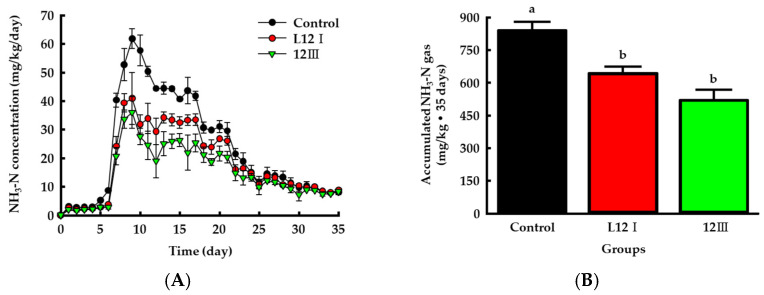
Changes of daily NH_3_ gas emission (**A**) and accumulated NH_3_ gas (**B**) in the livestock manure by treating selected bacterial strains. Values represent the mean ± S.E. (*n* = 3). The different letters (a, b) indicate statistically significant difference between groups (significance level at *p* < 0.05).

The emission pattern of ammonia from manure due to the treatment with selected strains was tested using a gas trapping device and an ammonia quantification method. Overall, the emission pattern was observed over 35 days in the experimental groups, and the pattern increased rapidly and then gradually decreased, similar to that reported for urea-derived ammonia emission from soils [36,52] (Figure 8A). Total accumulated ammonia emissions for the 35 days were 841.43 ± 38.36, 643.02 ± 31.05, and 521.71 ± 47.27 mg/kg in the control, L12I, and 12III groups, respectively (Figure 8B). The groups treated with L12I and 12III showed significant emission reduction effects of 23.58% and 38.00%, respectively, compared to the control group (*p* < 0.05).

Additionally, the chemical properties of the manure were investigated to determine how the environmental parameters of the manure changed due to the treatment with the selected strains during the discharge analyses (Table 4). Among the chemical indicators, OM and pH did not show any significant differences among all groups (*p* > 0.05), and the T-N of the control group decreased by 22.16% compared to the initial content of emission (at day 0). In contrast, L12I and 12III strains, which had an emission reducing effect, showed 14.93% and 10.27% T-N reduction rates, respectively; in particular, the 12III treatment group, which had the best ammonia emission reduction effect at 38.00%, also had the best nitrogen conservation capacity. In the case of urea reduction rate, the control, L12I, and 12III treated groups showed 62.18%, 45.57%, and 45.00% reduction, respectively. Consequently, it was confirmed that the change in urea content between groups showed similar patterns to the change in T-N content and ammonia emission between groups, suggesting that urea and T-N content were major factors affecting the emissions.

Livestock manure, as the main raw material for organic compost, is an important nitrogen source [11]. Ammonia emissions from manure not only cause nitrogen depletion [11] but also adversely affect manure quality. The PA strains selected in this study were proven to be beneficial bacterial strains that reduced ammonia emission by preserving the nitrogen content in manure and ultimately improving the manure quality as a fertilizer. It is difficult to maintain uniformity in manure environment because the distribution of intestinal microorganisms can steadily change according to the diet and activity of the host [53,54]. For this reason, the determination of the environmental changes, identified in this study as decisive factors for ammonia emission, was difficult. However, in this study, it was possible to verify that their ability had an effect on nitrogen fixation of manure and reduction in ammonia emission by treating L12I and 12III, which are selected strains with growth inhibition of the HAB strain and acidification ability in manure.

## 4. Conclusions

This study was conducted to investigate the cause of ammonia, a major factor of air pollution, emitted from livestock manure and to verify the efficacy of a novel microbial agent to reduce ammonia gas. L12I and 12III (*P. acidilactici*) were selected as effective strains to reduce pH, ammonia concentration, and urease activity, which act as emission factors. Based on the results of this study, it was verified that L12I and 12III are strains capable of acidifying manure and inhibiting the growth of HAB strains. Furthermore, it was confirmed that the selected strains with verified activity are unprecedented microbial agents with superior effects in reducing ammonia gas emitted from manure and fixing nitrogen for use as a fertilizer. Therefore, this study can be utilized as a basis for applying various control methods to reduce the emission of ammonia gas and contribute to the mitigation of air pollution derived from manure by utilizing the discovered microbial agents.

## Figures and Tables

**Table 1:** List of PCR primer pairs used for qPCR analysis.

Target Strain	Primer Name	Sequence (5′–3′)	Product Size (bp)	Reference
Total bacteria	UB-F	CGGCAACGAGCGCAACCC	161	[31]
	UB-R	CCATTGTAGCACGTGTGTAGCC		
*Pediococcus* *acidilactici*	IdhDF	GGACTTGATAACGTACCCGC	449	[32]
	IdhDR	GTTCCGTCTTGCATTTGACC		
*Clostridium* *aminophilum*	57F	ACGGAAATTACAGAAGGAAG	560	[33]
	616R	GTTTCCAAAGCAATTCCAC		
*Proteus mirabilis*	ureF	GGTGAGATTTGTATTAATGG	225	[34]
	ureR	ATAATCTGGAAGATGACGAG		

**Table 2:** Characteristics of carbohydrate fermentation patterns of selected bacteria.

Carbohydrates	L12 I	12 III	Carbohydrates	L12 I	12 III
Control	−	−	Esculin	−	+
Glycerol	−	−	Salicin	+	+
Erythritol	−	−	D-Cellobiose	+	+
D-Arabinose	−	−	D-Maltose	−	−
L-Arabinose	+	+	Lactose	−	−
D-Ribose	+	+	D-Melibiose	−	−
D-Xylose	+	+	Sucrose	−	−
L-Xylose	−	−	Trehalose	+	+
D-Adonitol	−	−	Inulin	−	−
Methyl-β D-xylopyranoside	−	−	D-Melezitose	−	−
D-Galactose	+	+	Raffinose	−	−
D-Glucose	+	+	Starch	−	−
D-Fructose	+	+	Glycogen	−	−
D-Mannose	+	+	Xylitol	−	−
L-Sorbose	−	−	Gentiobiose	−	+
L-Rhamnose	+	+	D-Turanose	−	−
Dulcitol	−	−	D-Lyxose	−	−
Inositol	−	−	D-Tagatose	+	+
Mannitol	−	−	D-Fucose	−	−
Sorbitol	−	−	L-Fucose	−	−
Methyl-α D-Mannopyranoside	−	−	D-Arabitol	−	−
Methyl-α D-Glucopyranoside	−	−	L-Arabitol	−	−
N-Acetylglucosamine	+	+	Potassium gluconate	−	−
Amygdalin	−	−	Potassium 2-ketogluconate	−	−
Arbutin	+	−	Potassium 5-ketogluconate	−	−

Symbols denote positive (+) and negative (−) in sugar utilization patterns of API 50 CHL test.

**Table 4:** Changes in chemical characteristics of manure samples treated with selected bacterial strains.

Group	Control		L12 I		12 III	
Time (Day)	0	35	0	35	0	35
OM (%)	41.66 ± 0.86	43.03 ± 1.06	42.69 ± 0.99	40.61 ± 0.54	42.21 ± 0.55	40.38 ± 1.16
T-N (%)	1.94 ± 0.05	1.51 ± 0.02 ^b^	2.01 ± 0.13	1.71 ± 0.16 ^b^	2.24 ± 0.10	2.01 ± 0.09 ^a^
OM/T-N ratio	21.51 ± 0.44 ^a^	28.47 ± 0.70 ^a,^*	21.26 ± 0.49 ^a^	23.70 ± 0.31 ^b,^*	18.88 ± 0.25 ^b^	20.12 ± 0.58 ^c^
Urea (mg/100g)	14.78 ± 1.04	5.59 ± 0.23 ^b,^*	15.29 ± 0.50	7.71 ± 0.12 ^a,^*	13.42 ± 0.24	7.38 ± 0.41 ^a,^*
pH	7.49 ± 0.02	8.46 ± 0.04 *	7.46 ± 0.02	8.49 ± 0.02 *	7.48 ± 0.02	8.48 ± 0.01 *

Values represent the mean ± S.E. (*n* = 3). *: statistical significance compared with 0 and 35 days of each group (*p <* 0.05) by using *t*-test. (a, b, c) letters: statistically significant difference between groups at the same day within a line (*p* < 0.05) by using one-way ANOVA. OM, organic matter; T-N, total nitrogen.

## Data Availability

All data are presented in the paper.
